# Exploring Factors Associated with Nonchange in Condom Use Behavior following Participation in an STI/HIV Prevention Intervention for African-American Adolescent Females

**DOI:** 10.1155/2012/231417

**Published:** 2012-05-29

**Authors:** Jessica M. Sales, Jennifer L. Brown, Ralph J. DiClemente, Eve Rose

**Affiliations:** ^1^Department of Behavioral Sciences and Health Education, Rollins School of Public Health, Emory University, Atlanta, GA 30322, USA; ^2^Center for AIDS Research, Social and Behavioral Sciences Core, Emory University, Atlanta, GA 30322, USA

## Abstract

To enhance future STI/HIV prevention efforts, this study examined factors associated with adolescents' failure to improve their condom use behaviors after participating in an STI/HIV prevention intervention. African-American adolescent females (*N* = 205; *M* age = 17.9) in an STI/HIV prevention intervention trial completed ACASI interviews and provided self-collected vaginal swabs to assess two prevalent STIs at baseline and 6 months after intervention. Analyses compared those who increased condom use after intervention (change group) to those whose condom use did not increase (nonchange group). 43.4% did not increase their condom use after the intervention and were more likely to have an STI at followup (*χ* ^2^ = 4.64, *P* = .03). In a multivariate logistic regression model, the nonchange group was more likely to have (a) higher sensation seeking (AOR = .91, *P* = .023), (b) a boyfriend (AOR = .32, *P* = .046), and/or (c) a physical abuse history (AOR = .56, *P* = .057). There were also differences in the extent to which psychosocial mediators changed between the two groups. Findings highlight the need to tailor STI/HIV interventions to adolescents with a greater degree of sensation seeking and address key relationship characteristics and trauma histories to bolster intervention efficacy.

## 1. Introduction

Adolescents and young adults are disproportionately affected by sexually transmitted infections (STIs) [1]. Among young people, girls have particularly high rates of STIs. Findings from the Centers for Disease Control and Prevention (CDC) indicate that, overall, one in four in the United States has an STI, with nearly half (48%) of African-American girls detected with an STI [2]. To combat the STI epidemic among adolescent females, several STI/HIV prevention interventions have been developed, including interventions designed to be culturally congruent for African-American adolescents and/or young adult females [3–9].

Typically, STI/HIV prevention interventions targeting adolescent females focus on increasing participants' knowledge regarding STI/HIV transmission dynamics, condom use self-efficacy, barriers to condom use, attitudes toward condom use, intentions to use condoms, condom use skills, and facilitating partner communication about sex and condom use [3–8]. A recent meta-analysis supports the inclusion of these components in interventions specifically tailored for African-American females: HIV prevention intervention efficacy was greatest in studies that specifically targeted African-American females, used gender- and/or culturally specific materials, addressed empowerment issues, provided skills training in condom use and negotiation of safer sex, and used role playing to teach negotiation skills [10]. Thus, a strong emphasis on enhancing communication and negotiation strategies may be particularly salient for African-American females within the context of STI/HIV prevention interventions.

Recent reviews of randomized controlled STI/HIV risk-reduction intervention trials suggest that tailored interventions for African-American adolescent females inclusive of all of the above-mentioned components (e.g., the Horizons intervention [5]) have repeatedly shown positive outcomes on key behavioral outcomes (e.g., consistent condom use) and also reduce STIs [9, 11, 12]. Although effective interventions to reduce STIs and risk behaviors exist, they are most potent in the short term and are not uniformly effective [13–15]. Thus, not everyone exposed to an STI/HIV risk-reduction intervention will positively change (i.e., reduce) their STI/HIV-associated risk behavior after participation in the intervention.

Recent studies conducted with high-risk adults have recognized the variability of initial and sustained responses to sexual risk reduction interventions [13–15]. They have demonstrated that even homogeneous subgroups show different patterns of change in response to tailored interventions [13, 14]. For instance, Kalichman et al. [13] examined patterns of sexual behavior change among adult STI clinic patients who received risk reduction counseling and were subsequently followed for 9 months after counseling. Cluster analyses identified three subgroups: (1) sustained low-risk behaviors over time, (2) significant reduction in risk behaviors over time, and (3) increased risk behaviors over time. However, no baseline behaviors differentiated the subgroups patterns of risk taking over time after intervention.

To date, understanding factors associated with nonresponsiveness to STI/HIV prevention interventions has been examined only in a very limited number of studies with adult populations. Little is known about the factors associated with adolescents' failure to reduce high-risk behaviors following participation in an STI/HIV prevention intervention. Thus, this study sought to explore factors associated with adolescent African-American females' failure to change their condom use behaviors after intervention. Specifically, we examined the extent to which sociodemographic, psychosocial, and life history factors assessed at baseline (prior to intervention participation) differed between those who increased their condom use from those who did not increase condom use after participating in the HORIZONS STI/HIV prevention intervention [5]. The Horizons intervention, guided by social cognitive theory [16] and the theory of gender and power [17, 18], targeted several constructs including fear of condom negotiation, partner communication self-efficacy, partner communication frequency, refusal self-efficacy, condom use self-efficacy, and STI knowledge. We examined the extent to which young women who either increased or did not increase condom use after intervention differed in regards to changes in the targeted psychosocial mediators from baseline levels. Such knowledge would be useful for the creation, revision, or adaptation of sexual risk reduction interventions for this especially vulnerable subgroup of nonresponsive adolescents.

## 2. Materials and Methods

### 2.1. Participants

Participants were part of a larger study evaluating a sexual risk reduction intervention tailored for African-American adolescents. From March 2002 to August 2004, African-American adolescent females, 15–21 years, were recruited from three clinics in downtown Atlanta, Georgia, providing sexual health services to predominantly inner-city adolescents. A young African-American woman recruiter approached all adolescents in the clinic waiting area, described the study, solicited participation, and assessed eligibility. Eligibility criteria included self-identifying as African-American, 15–21 years, and reporting vaginal intercourse in the past 60 days. Adolescents who were married, currently pregnant, or attempting to become pregnant were excluded from the study. Written informed consent was obtained from all adolescents with parental permission waived for those younger than 18 due to the confidential nature of clinic services. Of the eligible adolescents, 84.4% (*N* = 715) enrolled in the study, completed baseline assessments, and were randomized to study conditions. Regarding retention, 610 (85.3%) completed the 6-month assessment, and 605 (84.6%) completed the 12-month assessment. The main reason for participant loss over followup was because of moving or scheduling conflicts (see detailed description of study procedures in DiClemente et al., 2009) [5]. Participants were compensated $50 for each assessment, and the Emory University Institutional Review Board approved all study protocols.

### 2.2. Study Design and Data Collection

The study used a 2-arm randomized controlled trial design. Assignment to study conditions was implemented subsequent to baseline assessment using concealment of allocation procedures, defined by protocol and compliant with published recommendations. Prior to enrollment, investigators used a computer algorithm to generate a random allocation sequence and opaque envelopes to execute the assignments. 

Data collection occurred at baseline, 6 and 12 months following completion of the two-session group-implemented HIV/STD intervention and consisted of an audio computer-assisted self-interview (ACASI) and self-collected vaginal swab to assess two prevalent STIs. The ACASI assessed sociodemographics, alcohol and drug use, history of abuse, relationship status, sexual behaviors, and psychosocial correlates of risky sexual behavior (e.g., depression, self-esteem, sexual sensation seeking).

### 2.3. Intervention Methods

The intervention (HORIZONS) [5], which was effective in reducing risky sexual behaviors and STIs, consisted of three components: (1) administration of two 4-hour group STI/HIV prevention sessions, (2) provision of vouchers to participants to give to their male sexual partners to facilitate access to STI screening/treatment, and (3) the administration of 4 brief telephone contacts to reinforce prevention information presented in group sessions. The two 4-hour group sessions were facilitated by trained African-American women health educators. 

In the randomized controlled trial, the HORIZONS intervention arm was evaluated relative to a standard of care condition. The enhanced usual care comparison condition was a 1-hour group session, implemented by an African-American woman health educator, consisting of a culturally and gender-appropriate STI/HIV prevention video, a question-and-answer session, and a group discussion. Participants also received telephone contacts, on the same schedule as intervention participants, but only to update locator information, no additional STI/HIV prevention education was provided.

### 2.4. Measures

#### 2.4.1. Nonresponsiveness Status

As part of the main trial's assessments, percentage condom use during the previous 14 days was assessed by calculating the number of times condoms were used divided by the number of times the participant had intercourse. At each assessment, participants were asked, “In the past 14 days, how many times did you have vaginal sex?” Following this question, participants were asked, “Out of the XX times you've had vaginal sex, in the past 14 days, how many times did you use a condom?” Change in condom use was calculated by comparing participants' reported percentage condom use in the 14 days prior to baseline (prior to intervention participation) to their reported percentage of condom use in the 14 days prior to the six-month followup assessment (after intervention). If a participant reported any increase in their condom use from baseline levels, this was considered as “change” (coded as 1); if she reported no increase or a decrease in condom use, this was considered as “nonchange” (coded as 0). Condom use at the first six-month followup interval after intervention was selected because most HIV risk-reduction programs see the strongest impact on behavior change (i.e., increased condom use) closer to intervention participation, with effects tending to wane over time [19]. The “change group” and “nonchange group” did not significantly differ in their baseline rates of condom use.

#### 2.4.2. Sociodemographics

**Age and Current School Attendance**

Participants completed questions regarding their age and whether they were currently attending school.

**Neighborhood Quality**

Neighborhood quality was assessed with 3 questions about the physical condition of participants' neighborhood. A sample item is “On your street, are there abandoned homes or apartments?” Responses to all three Yes/No questions were summed to create an index of neighborhood quality with higher scores indicative of poorer neighborhood quality [20].

**Family Aid**

Family aid was assessed with 4 questions about receipt of family aid in the form of money or services during the past 12 months (e.g., Section  8 housing, food stamps). Responses to the four Yes/No questions were summed to create an index of total family aid.

#### 2.4.3. Life History Factors

**Substance Use History**

Separately, participants self-reported whether they had ever used (a) alcohol or (b) marijuana. Response choices for each question were yes (1) or no (0).

**Abuse History**

Separately, participants self-reported whether they had previously experienced any (a) physical abuse or (b) sexual abuse (e.g., forced vaginal sex). Response choices for each question were yes (1) or no (0).

**Current Relationship Status**

Current relationship status was assessed by asking participants whether they had a boyfriend. Response choices were yes (1) or no (0).

**Sexually Transmitted Infections**

Participants provided a self-collected vaginal swab specimen [21]. Specimens were delivered to the Emory University Pathology Laboratory where the specimen was assayed for* C. trachomatis* and *N. gonorrhoeae*. Initially, *C. trachomatis* and *N. gonorrhoeae* were assayed using the Abbott LCx Probe System (Abbott Laboratories, Abbot Park, IL, USA) [22, 23]. In September of 2002, this assay was discontinued, and all subsequent testing used the BDProbeTec ET *C. trachomatis* and *N. gonorrhoeae* Amplified DNA assay (Becton Dickinson and Company, Sparks, MD) [24]. Participants with a positive STI test received directly observable single-dose treatment and risk-reduction counseling per CDC recommendations and were encouraged to refer sex partners for STI screening and treatment. The County Health Department was notified of reportable STIs, which included both *C. trachomatis* and *N. gonorrhoeae*. Those testing positive for one or both STI were coded as (1) and those who were negative for both were coded as (0).

#### 2.4.4. Psychosocial Factors

**Sexual Sensation Seeking**

Sexual sensation seeking was assessed by a 9-item scale [25]. Sample items include “When it comes to sex, I'm willing to try anything” and “Stopping to use a condom during sex takes the fun out of sex.” Participants rated each item from 1 (strongly disagree) to 4 (strongly agree), with higher scores indicating higher levels of sensation seeking. Cronbach's alpha at baseline was 0.72.

**Depressive Symptoms**

Respondents' depressive symptoms were measured with the 8-item Center for Epidemiological Studies-Depression scale (CES-D) [26]. The CES-D assesses the frequency of depressive symptoms experienced in the past 7 days from less than one day (1) to five to seven days (4) with higher scores indicative of greater frequency of depressive symptoms. Cronbach's alpha was 0.89.

**Social Support**

Perceived social support was assessed with an 11-item scale [27]. A sample scale item includes “I can count on my friends when things go wrong.” Participants rated each item from 1 (strongly disagree) to 4 (strongly agree), with higher scores indicating higher levels of perceived social support. Cronbach's alpha was 0.90.

**Self-Esteem**

The Rosenberg self-esteem Scale, a 10-item scale, measured global self-esteem [28]. A sample item from the scale is “I feel that I am a person of worth” with responses ranging from strongly disagree (1) to strongly agree (4). Possible scores range from 10 to 40, with higher scores indicating higher levels of self-esteem. Cronbach's alpha was 0.85.

**Locus of Control**

A 4-item scale assessed locus of control [29]. Participants rated each item from strongly disagree (1) to strongly agree (4); a sample item is “I have little control over the things that happen to me.” Higher scores on the measure indicate a greater tendency towards an external locus of control. Cronbach's alpha was 0.63.

**Perceived Peer Norms**

Eight items assessed perceived peer norms supporting risky sexual behavior [30]. Using a five-point scale (1: “none” to 5: “all”), participants were asked to report the number of same age peers who engaged in each sexual risk behavior (e.g., “have sex with someone you just met”). Higher scores indicated greater perceived peer norms supporting risky sexual behaviors. Cronbach's alpha was 0.68.

#### 2.4.5. Psychosocial Mediators Targeted in the HORIZONS Intervention

All of the following measures have been successfully used in prior studies with African-American adolescent females and achieved adequate levels of reliability [4, 5].

**Condom Use Self-Efficacy**

Nine items measured participants' self-efficacy to use condoms correctly [31]. For each item, participants indicated how much of a problem it would be to use a condom using a five-point scale (i.e., “none,” “not much,” “a little,” “some,” and “a lot”). Items were summed with higher scores indicating greater condom use self-efficacy. Cronbach's alpha was 0.86.

**Partner Communication Self-Efficacy**

Self-efficacy to communicate with partners was assessed by a 6-item measure [31]. For each item, participants indicated how difficult it would be to communicate with a partner about sexual health topics and condom use (e.g., “How hard is it for you to ask if he has an STD?”) using a four-point scale (i.e., “very hard,” “hard,” “easy,” and “very easy”). Higher scores were indicative of greater partner communication self-efficacy. Cronbach's alpha was 0.83.

**Partner Communication Frequency**

Five items measured the frequency of sexual communication with one's sexual partner [32]. For each item, participants rated the frequency they had discussed each of the topics (e.g., strategies to prevent getting STDs) with their partner during the past 60 days using a 4-point scale (i.e., “never,” “1–3 times,” “4–6 times,” and “7 or more times”). Higher scores on the measure indicated greater sexual communication frequency. Cronbach's alpha was 0.84.

**Sex Refusal Self-Efficacy**

Self-efficacy to refuse unwanted sexual activity was assessed using a 7-item measure [33]. A sample item included “How sure are you that you would be able to say no to having sex with someone you have known for a few days for less.” Response options ranged from “I definitely cannot say no” (1) to “I definitely can say no” (4). Responses were coded such that higher scores indicated greater self-efficacy to refuse unwanted sexual activity. Cronbach's alpha was 0.87.

**Fear of Condom Negotiation**

Fear of consequences of condom negotiation with a sexual partner was assessed by an 8-item scale [31]. Sample consequences for negotiated condom use were “ignore my request,” “hit, push or kick me,” “leave me,” and “go out with other girls.” Higher scores indicated greater fear of communicating about condoms with a partner. Cronbach's alpha was 0.84.

**STI Knowledge**

STI knowledge was measured with an 11-item index [34]. For each statement, participants indicated whether the statement was “true” or “false.” If a participant did not know, she was instructed to respond with “do not know.” Each item was scored for correctness (“do not know” was scored as an incorrect response). A total score was calculated where higher scores indicated greater STI knowledge.

### 2.5. Data Analyses

All analyses were limited to HORIZONS participants randomized to the intervention arm who also returned for the six-month followup assessment (postintervention participation). Because of this, coupled with the use of ACASI for assessment, we had no missing data from participants at baseline and the 6-month followup assessments. Descriptive statistics summarized intervention responsiveness rates. In addition, analyses examined differences between groups (nonchange group versus change group) on sociodemographic variables, psychosocial characteristics, and life history factors reported at the baseline assessment (prior to intervention participation). Differences were assessed using independent samples *t*-tests for continuous variables and chi-square analyses for categorical variables. Variables significant at the *P* ≤ .10 in bivariate analyses were entered into a multivariable logistic regression predicting change status at the six-month followup assessment. Additionally, a repeated measure MANOVA was conducted, with behavioral change status entered as a between-subjects factor (nonchange group versus change group), and each of the six targeted psychosocial mediators entered as repeated-measures within-subjects factors (baseline value of psychosocial mediator and six-month assessment value of psychosocial mediator) to examine the extent to which change in mediators differed as a function of behavioral change status. The significant model was followed by separate univariate repeated measures ANOVAs for each mediator.

## 3. Results

### 3.1. Sample Description

Mean age of participants in this sample (*N* = 205) was 17.9 years (SD = 1.61) upon enrollment into the main trial. The majority was still enrolled in school (62.9%). Thirty percent had a job, and many reported living with their mother only (41.0%) or mother and father (14.6%). Condoms were used, on average, 48% of the vaginal sex occasions in the 14 days prior to baseline assessment and 18% tested positive for either chlamydia and/or gonorrhea at baseline. 

Of key interest for this study, among the 205 intervention participants who provided information on recent condom use at both baseline and 6-month followup assessment, 43.4% (*n* = 89) did not increase their condom use from baseline levels six months after participating in a culturally and gender-tailored STI/HIV prevention intervention. Importantly, this trial demonstrated efficacy in reducing sexual risk behaviors (i.e., increasing condom use in the 14 days prior followup assessment) and incident STIs among adolescent African-American girls (i.e., chlamydia and gonorrhea infections) [5]. Additionally, we found a significant difference between the nonchange and change groups (*χ* ^2^ = 4.64, *P* = .03) for STI incidence at the 6-month followup assessment, such that adolescents in the nonchange group had greater odds of testing positive for either chlamydia and/or gonorrhea at followup than adolescents in the change group (21.3% versus 10.4%, resp.).

### 3.2. Bivariate Associations among Study Variables

Behavioral change groups were compared in regards to several sociodemographic, life history, and psychosocial characteristics at the baseline assessment (see Table 1). Compared to the change group (i.e., adolescents who increased their recent condom use from baseline levels at the 6-month followup), those in the nonchange group had (a) greater odds of trying marijuana in their lifetime, (b) greater odds of a physical abuse history, (c) greater odds of having a current boyfriend, and/or (d) reported higher levels of sexual sensation seeking. Additionally, behavioral change groups were compared in regards to the baseline values of the psychosocial mediators targeted in the intervention (see Table 3 for means/SDs by group at baseline). The only significant group difference between the baseline values of the psychosocial mediators was for the sexual refusal self-efficacy, *t*(203) = −2.18, *P* = .04.

### 3.3. Multivariable Logistic Regression Predicting Behavioral Change Status Postintervention Participation

Factors identified as significant in bivariate analyses were entered into a multivariable logistic regression, controlling for age, to determine which factors were significantly associated with behavioral change status 6 months after participating in the HORIZONS intervention workshops (see Table 2). Adolescents reporting higher levels of sexual sensation seeking and individuals with a current boyfriend at baseline were at significantly greater odds of nonchange in recent condom use after intervention compared to those with lower levels of sexual sensation seeking and adolescents without a boyfriend. Additionally, adolescents reporting a history of physical abuse at baseline were at marginally significant greater odds of nonchange in recent condom use after intervention (*P* = .057).

### 3.4. Change in Psychosocial Mediators after HORIZONS Participation

A 2 × 2 repeated measures MANOVA was conducted including all six psychosocial mediators as outcomes in the model. Behavioral change status (nonchange group versus change group) was the between-subject factor in the model, and time (baseline value of mediator versus followup value of mediator) was a repeated measure, within-subject factor in each model. Means and standard deviations for all six psychosocial mediators targeted in HORIZONS at baseline (prior to intervention) and, again, at 6-month followup assessment (after intervention) are presented in Table 3. There was an overall significant main effect of time, *F*(6,198) = 15.74, *P* < .001, and a significant interaction between time and behavioral change status, *F*(6,198) = 2.52, *P* = .02. Separate univariate repeated measures ANOVAs were performed for each psychosocial mediator. 

For condom use self-efficacy, we found an overall significant main effect for time *F*(1,203) = 36.41, *P* < .001, with increased condom use self-efficacy scores post-intervention (*M* = 15.01, SD = 6.28) compared to baseline (*M* = 12.42, SD = 4.38). No other significant main effects or interactions were found. In regards to partner communication self-efficacy, we found an overall significant main effect for time, *F*(1,203) = 6.48, *P* = .01, with improved partner communication self-efficacy scores after intervention. This main effect should be interpreted within the context of a marginally significant interaction between time and behavioral change status, *F*(1,203) = 3.03, *P* = .08. Paired-sample *t*-tests were conducted separately for each behavioral change group to followup this interaction and revealed that the nonchange group's partner communication self-efficacy scores did not increase after intervention, *t*(88) = −0.5, *P* = .58, but there was a significant improvement over time for the change group, *t*(115) = −3.17, *P* = .002. For partner communication frequency, there were no significant main effects or interactions. For sexual refusal self-efficacy, we found a significant interaction between time and behavioral change status, *F*(1,201) = 8.76, *P* = .003. Paired-sample *t*-tests were conducted separately for each behavioral change group to further examine this interaction and revealed that the nonchange group's refusal self-efficacy scores increased after intervention, *t*(88) = −2.97, *P* = .004, but there was no difference over time for the change group, *t*(115) = 1.16, *P* = .25. Finally, for fear of condom use negotiation, we found a main effect of behavioral change status, *F*(1,203) = 4.58, *P* = .03, such that the nonchange group reported significantly higher fear of condom negotiation scores (*M* = 10.79, SD = 4.03) than the change group (*M* = 9.75, SD = 2.94). There was no significant interaction. For STI knowledge, we found an overall significant main effect for time *F*(1,203) = 40.66, *P* < .001, with increased STD knowledge scores after intervention (*M* = 19.07, SD = 2.20) compared to baseline (*M* = 18.14, SD = 1.77). No other significant main effects or interactions were found.

## 4. Discussion

Similar to the limited findings reported in the adult HIV prevention literature on nonchange in preventive behaviors after intervention [13], nearly half of adolescents who participated in a demonstrated efficacious STI/HIV risk-reduction program did not increase their condom use after intervention. Those reporting no increase in recent condom use at the six-month followup assessment were more likely to have higher levels of sensation seeking. In addition, adolescents whose condom use did not improve were more likely to have a current boyfriend and a physical abuse history. Additionally, those who did not increase their recent condom use after intervention reported higher levels of fear of condom negotiation across time and no significant increase in partner communication self-efficacy from baseline levels after intervention participation. 

Our findings are consistent with research examining the relationship between sensation seeking and sexual risk taking [35]. However, our findings extend this literature by demonstrating that individuals with higher levels of sexual sensation seeking are at increased odds of not increasing their condom use behavior after participating in a demonstrated efficacious STI/HIV prevention intervention. Research has shown that people high on sensation seeking tend to evaluate risky activity as less risky than those with low or moderate levels of sensation seeking [36, 37]. Moreover, research on the association between sensation seeking and message manipulation suggests that it may be beneficial to alter intervention strategies to affect the sexual risk taking of high-sensation seekers because they respond to stimuli differently than low-sensation seekers [38]. Specifically, high-sensation seekers tend to require stronger, novel, and highly arousing messages to hold their attention, whereas low-sensation seekers tend to respond better to more familiar and less intense stimulation. Additionally, high-sensation seekers are more likely to engage in risky behavior to seek out or enhance pleasure, whereas low-sensation seekers might participate in risky behavior for different reasons including the desire to be liked or fit in with one's peers [39]. Thus, future STI/HIV prevention efforts for adolescents may consider screening for sensation seeking tendencies and testing alternate styles of presenting intervention content to best reach youth with higher levels of sensation seeking. 

Adolescents who were in a current relationship with a boyfriend were also less likely to increase their condom use after participating in the intervention. Young women who are in established relationships where condoms have not been consistently used may have greater difficulty negotiating future condom use with their partners [40]. Additionally, it may be that young women with higher-sensation seeking levels select sexual partners that pose greater risks for adverse sexual health outcomes (e.g., partners with STI history) [36, 37]. These relationships may also reflect a power imbalance between partners, such that young women have less ability to negotiate condom use [17, 18]. Future studies should further examine the extent to which specific partner characteristics impact adolescents' responsiveness to sexual health programming. STI/HIV prevention interventions may benefit from providing specific strategies to negotiate condom use in established partnerships where condom use is not normative. Additionally, young women who do not improve recent condom use after participating in an STI/HIV prevention intervention may also need further individualized contact to address specific barriers that exist within the context of their current relationship. 

According to the theory of gender and power [17, 18], the experience of abuse disempowers women to negotiate safer sexual practices in their current sexual relationships because they may fear possible ramifications by male partners [41–43]. The findings of this study provide some support for this position by demonstrating that young women reporting a history of physical abuse are at marginally greater odds of not increasing their recent condom use after intervention. Furthermore, young women who did not increase their condom use after participating in HORIZONS reported higher overall levels of fear of negotiating condom use in general, as well as no significant increase in partner communication self-efficacy scores after intervention. Thus, similar to successful interventions designed to reduce traumatic stress and sexual risk among people living with HIV who have histories of abuse [44, 45], STI/HIV prevention interventions for young women may consider providing more in-depth discussion and instruction on specific strategies to manage and overcome fear or anxiety related to past abuse, as well as fear/anxiety about being assertive in current sexual situations. Doing so may improve the efficacy of STI/HIV prevention programs for those who have a history of abuse. 

Many STI/HIV prevention programs designed for young women, including HORIZONS [5], include a small component on unhealthy relationships (i.e., identifying abusive relationships). However, they may not thoroughly address the multitude of additional factors stemming from prior abuse (i.e., fear and anxiety) that may be hindering young women from living both emotionally and physically healthy lives. Thus, interventions should increase awareness of the co-occurrence of abuse, fear, and sexual risk behaviors, focus on developing cognitive and behavioral skills needed to accurately appraise risk, identify triggers associated with negative affect and sexual risk-taking, and develop strategies to avoid situations that trigger engaging in sexual risk taking. 

Specific to the psychosocial mediators targeted in HORIZONS, overall we found that regardless of whether participants did or did not increase their condom use behavior after intervention, they did increase their STI knowledge and condom use skills after participating in the HORIZONS workshops. These findings suggest that adolescents benefited from the specialized activities targeted to increase knowledge and condom use skills in the workshop. However, increases in partner communication self-efficacy scores over time were primarily observed for those that increased their condom use over time. This suggests that, again, partner-level factors may hinder young women from feeling self-efficacious to communicate about sexually related topics, including condom use, with their partners. We did observe significant increases in sexual refusal self-efficacy after intervention but only for the nonchange group. This finding is counterintuitive and requires further exploration, but it should be noted that the nonchange group's followup levels of sexual refusal self-efficacy were comparable to the baseline levels of the change group.

## 5. Limitations

This study is not without limitations. First, the data employed in this study were only from participants who returned for the followup assessment after the intervention workshop. Thus, it is possible that young women who did not return for their followup assessment may differ in meaningful ways from women who did return for followup, but we have no way to formally examine this possibility. However, analyses of baseline sociodemographic characteristics and behaviors indicate no significant differences between those who returned for followup and those who did not. Finally, the findings of this study may not generalize to other young women who have participated in STI/HIV prevention programs, especially if they participated in programs that varied markedly in content from HORIZONS.

## 6. Conclusion

Several efficacious STI/HIV prevention programs exist for a variety of populations, including African-American adolescent girls [9, 46]. Despite the demonstrated efficacy of interventions to reduce STI/HIV-associated sexual risk behaviors, not every individual exposed to such a program will positively change their sexual risk behaviors (i.e., increase condom use) following participation in an STI/HIV risk-reduction intervention. The ability to identify barriers and possible causal factors that differentiate those who increased condom use after intervention from those who did not is the first step in refining, adapting, or designing new STI/HIV prevention programs to optimize their appropriateness and efficacy for these especially vulnerable youth.

## Figures and Tables

**Table 1:** Group differences in sociodemographic, life history, and psychosocial characteristics assessed at baseline assessment (*N* = 205).

Study variables	Nonchange group (*n* = 89)	Change group (*n* = 116)	Test statistic	*P*
*Sociodemographic*				
Age^b^	18.01 (1.66)	17.83 (1.59)	0.77	.44
Neighborhood quality^b^	0.51 (.79)	0.63 (.95)	−0.98	.32
Family aid index^b^	0.82 (.98)	0.76 (.88)	0.47	.64
Currently attending school^a^	52 (58.4)	77 (66.4)	1.37	.24

*Life history*				
Ever tried alcohol^a^	77 (86.5)	96 (82.8)	0.54	.46
Ever tried marijuana^a^	79 (88.8)	91 (78.4)	3.79	.05
History of sexual abuse^a^	24 (27.0)	26 (22.4)	0.57	.45
History of physical abuse^a^	52 (58.4)	48 (41.4)	5.86	.02
Current boyfriend^a^	84 (94.4)	101 (87.1)	3.06	.08
Positive laboratory confirmed STI^a^	16 (18.0)	21 (18.1)	.001	.98

*Psychosocial factors*				
Sexual sensation seeking^b^	18.35 (3.72)	16.78 (3.77)	2.96	.003
Self-esteem^b^	33.06 (5.36)	33.63 (5.02)	−0.79	.43
Locus of control^b^	7.28 (2.45)	7.32 (2.39)	−0.11	.91
Depressive symptomatology^b^	16.33 (6.57)	16.79 (7.43)	−0.46	.65
Social support^b^	34.53 (7.11)	34.68 (7.45)	−0.15	.88
Peer norms^b^	20.99 (4.72)	20.15 (5.02)	1.21	.23

Note: ^a^frequency (%) presented, and test statistic is chi-square; ^b^mean (SD) presented, and test statistic is *t*-test.

**Table 2:** Multivariable regression prediction behavioral change status 6 months after participating in HORIZONS.

			95% CI			
Predictor	*β*	SE	Odds ratio	Lower	Upper	*P*
*Sociodemographic*						
Age	−.075	.10	.93	.771	1.12	.431

*Life history*						
Ever tried marijuana	−.648	.42	.523	.230	1.19	.123
Current boyfriend	−1.142	.57	.319	.104	.978	.046
History of physical abuse	−.573	.30	.564	.312	1.01	.057

*Psychosocial factor*						
Sexual sensation seeking	−.094	.04	.91	.839	.987	.023

Overall *χ* ^2^ =	19.65					.001

**Table 3:** Means (and standard deviations) for psychosocial mediators targeted in HORIZONS at baseline and 6-month followup assessment, by behavioral change group (*N* = 205).

Psychosocial mediators	Baseline		Six-month followup	
	Nonchange group	Change group	Nonchange group	Change group
Condom use self-efficacy	15.25 (7.01)	14.82 (5.68)	13.07 (4.96)	11.93 (3.81)
Partner communication self-efficacy	20.85 (3.31)	21.07 (3.62)	21.04 (3.01)	22.03 (2.74)
Partner communication frequency	11.84 (4.35)	11.90 (4.63)	11.79 (4.48)	12.03 (4.64)
Sexual refusal self-efficacy	23.96 (4.79)	25.16 (3.16)	25.52 (3.42)	24.61 (4.63)
Fear of condom negotiation	10.57 (4.62)	10.03 (3.49)	11.01 (5.60)	9.47 (3.91)
STI knowledge	18.11 (1.92)	18.16 (1.65)	19.10 (2.27)	19.04 (2.16)
